# Association between egg consumption and arterial stiffness: a longitudinal study

**DOI:** 10.1186/s12937-021-00720-6

**Published:** 2021-07-13

**Authors:** Naiwen Ji, Zhe Huang, Xinyuan Zhang, Yuanyuan Sun, Shumao Ye, Shuohua Chen, Katherine L. Tucker, Shouling Wu, Xiang Gao

**Affiliations:** 1 Department of Nutritional Sciences, The Pennsylvania State University, 109 Chandlee Lab, University Park, State College, PA 16802 USA; 2 Department of Cardiology, Kailuan General Hospital, Tangshan, People’s Republic of China; 3 Department of Epidemiology and Biostatistics, Peking Union Medical College, Beijing, China; 4 Cardiovascular Nutrition Laboratory, Jean Mayer USDA Human Nutrition Research Center on Aging at Tufts University, Boston, USA; 5 Health Care Center, Kailuan General Hospital, Tangshan, China; 6 Department of Biomedical and Nutritional Sciences, University of Massachusetts, Lowell, MA USA

**Keywords:** Egg consumption, Arterial stiffness, Cardiovascular disease, Longitudinal study

## Abstract

**Background:**

Inconsistent associations between egg consumption and cardiovascular disease (CVD) risk have been observed in previous studies. This study aims to longitudinally investigate the association between egg consumption and altered risk of arterial stiffness, a major pre-clinical pathogenic change of CVD, which was assessed by brachial-ankle pulse wave velocity (baPWV).

**Methods:**

A total of 7315 Chinese participants from the Kailuan Study, free of CVD and cancer were included in this study. Egg consumption was assessed by a semi-quantitative validated food frequency questionnaire in 2014. baPWV was repeatedly measured at baseline and during follow-up (mean follow-up: 3.41 years). General linear regression was used to calculate means of baPWV change rate across different egg consumption groups, adjusting for age, sex, baseline baPWV, healthy eating index, total energy, social-economic status, blood pressure, obesity, smoking, lipid profiles, and fasting glucose concentrations.

**Results:**

Compared to the annual baPWV change rate in participants with 0–1.9 eggs/wk. (adjusted mean: 35.9 ± 11.2 cm/s/y), those consuming 3–3.9 eggs/wk. (adjusted mean: 0.2 ± 11.4 cm/s/y) had the lowest increase in baPWV during follow-up (P-difference = 0.002). Individuals with low (0–1.9 eggs/wk) vs. high (5+ eggs /wk) egg intake showed similar changes in baPWV.

**Conclusions:**

In this large-scale longitudinal analysis, we did not find a significant difference in arterial stiffness, as assessed by baPWV level, between low and high egg consumption groups. However, moderate egg consumption (3–3.9 eggs/wk) appeared to have beneficial effects on arterial stiffness.

**Supplementary Information:**

The online version contains supplementary material available at 10.1186/s12937-021-00720-6.

## Background

Eggs are a type of inexpensive, nutrient-dense food, an excellent source of protein, and a good source of lutein/zeaxanthin, vitamins, and minerals [1]. Eggs are also high in other biologically active compounds with antimicrobial, immunomodulatory, antioxidant, anti-cancer properties. Besides, egg protein has the highest biological value for providing essential amino acids that stimulate skeletal muscle synthesis [2]. Eggs are also one of the major sources of dietary cholesterol in the human diet [3]. A meta-analysis with 55 studies reported that high dietary cholesterol intake was associated with elevated serum cholesterol [4]. A recent Chinese study also reported that high dietary cholesterol intake was associated with hypercholesterolemia [5]. However, inconsistent associations between egg consumption and CVD risk have been observed in previous studies, and no clear association between egg consumption and cardiovascular disease (CVD) risk has been established. Several recent systematic reviews and meta-analyses showed no significant association between egg consumption and CVD mortality [6, 7] or altered status of CVD risk factors [8, 9]. In contrast, a recent pooled analysis of prospective study reported that each additional 0.5 egg/day consumption was associated with a 6% higher risk of CVD and 8% higher risk of all-cause mortality [10].

With the purpose of better understanding the relation between egg consumption and CVD, it is important to understand whether egg intake is associated with altered risk of major pre-clinical pathogenic progressions of CVD. Arterial stiffness is recognized as an important consequence of aging that has been shown to provoke deleterious vascular phenotypes in diseases such as diabetes [11], atherosclerosis [12], kidney dysfunction [13], and cognitive impairment [14]. Pulse wave velocity (PWV) is currently considered the gold standard in the evaluation of arterial stiffness [15]. PWV assesses the velocity of the blood pressure wave as it travels a known distance between two anatomic sites within the arterial system, and indicates the elasticity and other properties of the artery [16]. Arterial stiffness, as assessed by Brachial-ankle pulse wave velocity (baPWV), has been shown to be associated with subsequent CVD risk and having lower PWV at mid-life was associated with a lower risk of developing age-related CVD risk [17]. We, thus, conducted analysis with data from a large-scale longitudinal study, to examine whether egg intake was associated with the change in PWV during 3 years of follow-up, in 7315 adults without CVD.

## Methods

### Study population

The data utilized are from the Kailuan Study, a population-based prospective cohort launched in 2006 in Tangshan, China (The Chinese Clinical Trial Registry #: ChiCTR-TNRC-1101489). Participants recruited in the study were followed via biennial face-to-face physical examination, anthropometry, laboratory assessment, and annual comprehensive surveillance of medical records and death certificates, as detailed previously [12, 18, 19].

**Fig. 1:**
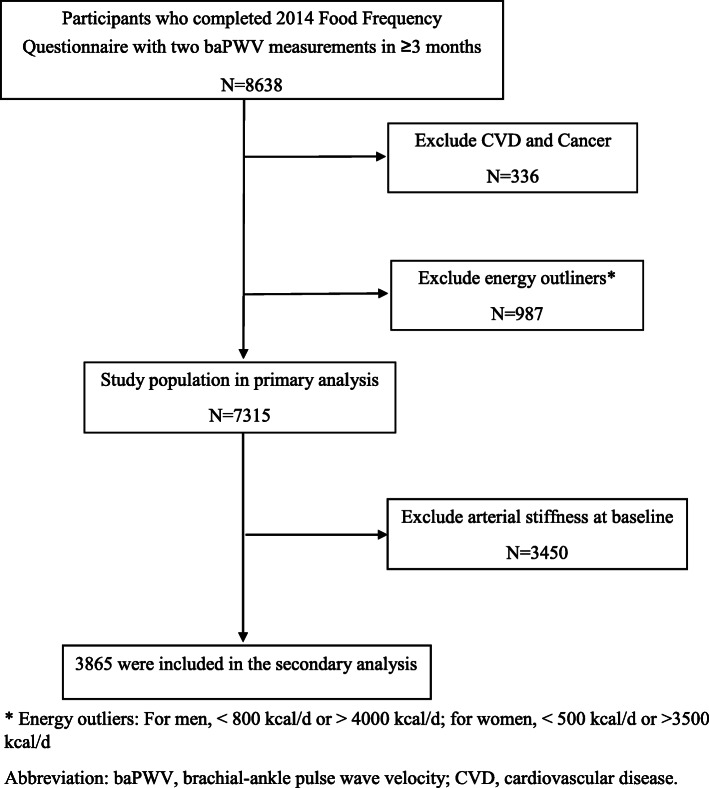
Flow chart of study participants selection. * Energy outliers: For men, < 800 kcal/d or > 4000 kcal/d; for women, < 500 kcal/d or > 3500 kcal/d. Abbreviation: baPWV, brachial-ankle pulse wave velocity; CVD, cardiovascular disease

BaPWV measurement was introduced into the testing routine in 2010 and repeatedly measured in later rounds for a sub-cohort of the Kailuan study, as described previously [12, 20]. BaPWV was measured for each participant at least twice between 2010 and 2016 and the interval between two measurements was more than 3 months. (mean difference between two assessments = 3.41 years). Dietary data were collected in 2014 (baseline for the current analysis), and 8638 participants aged 22 years or older (mean age: 48.6 ± 10.8 years) had completed dietary information and two baPWV assessments at and after the baseline. We further excluded 336 participants with CVD or cancer and 987 participants with energy intake out of range (for men, < 800 kcal/d or > 4000 kcal/d; for women < 500 kcal/d or > 3500 kcal/d; Fig. 1), leaving a total of 7315 participants in the current analysis. The primary outcome variable was the longitudinal change in baPWV (mean follow-up: 3.41 years; interquartile range:1.99–5.13 years). In the secondary analysis for incident arterial stiffness, 3865 participants without arterial stiffness (baPWV< 1400 cm/s) at baseline were included. The study was performed according to the guidelines of the Helsinki Declaration and was approved by the Ethics Committee of the Kailuan General Hospital.

### Assessment of egg consumption and other dietary components

In 2014, a 40-item (33 food items and 7 condiments) semi-quantitative food frequency questionnaire was used to collect possible food items participants consumed, which was validated in the Chinese population previously [21]. The food frequency questionnaire was administered along with other assessments during the face-to-face interview [14]. For each food item, participants were asked to provide information regarding the frequency (never, daily, weekly, or monthly) and quantity (liangs, 1 liang =50 g) of their consumption.

Specifically, a question regarding egg consumption was included in this food frequency questionnaire: “Based on your recent 1-year diet, do you usually eat egg including chicken egg, duck egg, etc.?” Participants answered this question by selecting the following options, “very often, __times/day”, “often, __times/week”, “sometimes, __times/month”, “never” and participants were then asked to provide how many eggs they consumed each time, on average. Based on self-reported egg consumption, we grouped the participants into 5 categories (0–1.9, 2–2.9, 3–3.9, 4–4.9, or ≥ 5 eggs/wk).

Overall diet quality, a potential confounder that might be associated with both egg consumption and arterial stiffness status, was evaluated via the dietary approaches to stop hypertension (DASH) score. The DASH score was focused on food and nutrients emphasized or minimized in the DASH diet, including nine components: vegetables, fruit, dairy, beans, whole grains, meat, fat, sodium, and sweetened beverage. Component scores were assigned to each participant quintile according to their intake ranking. As for vegetables, fruits, dairy, beans, and whole grains, the highest intake ranking quintile was assigned five points and the lowest intake ranking quintile was assigned one point. For sodium, meat, fat, and beverage, the lowest quintile was given a score of five points and the highest quintile was considered as one point. We then summed up the component scores to obtain an overall DASH score ranging from 9 to 45 [22, 23].

### Measurement of arterial stiffness

Arterial stiffness status was assessed at baseline and follow-up (mean 3.41y) using baPWV, which has been used in Asian populations [12, 24] and has previously been described [12, 20]. Briefly, participants were asked to refrain from smoking and to rest for at least 5 min before the measurement of baPWV. Cuffs were placed on both the brachial area of the arm and the ankle to assess the pulse transit time which is defined as the travel time of the pulse wave from brachia to ankle. In our study, baPWV was measured with a BP-203RPEIII networked arteriosclerosis detection device (OMRON Healthcare (China) Co., LTD.). The measurement was repeated twice at each visit to ensure accuracy, and the baPWV value was read by trained health professionals. Therefore, in this study, arterial stiffness was noted using a cut-off point of baPWV ≥1400 cm/s [17].

### Assessment of covariates

Baseline information was assessed with a validated questionnaire, administered in a face-to-face interview. The questionnaire included basic information (e.g. age, sex, marriage status, education level, smoking status, and occupation). Physical activity was assessed with the International Physical Activity Questionnaire (IPAQ) short form, which assesses the duration and frequency of sitting, walking, moderate-intensity activities, and vigorous-intensity activities, and has been validated in the Chinese population [25, 26].

Height, weight, and blood pressure were measured by trained health professionals, as detailed previously [12]. Body Mass Index was calculated as weight, in kilograms, divided by height, in meters, squared. Blood samples were collected the morning after a minimum of 8 h of fasting. Fasting blood glucose (FBG), high-density lipoprotein cholesterol (HDL-c), and low-density lipoprotein cholesterol (LDL-c) were measured in the central lab, Kailuan General Hospital, as previously described [12].

### Statistical analysis

All statistical analyses were performed in SAS version 9.4 (SAS Institute Inc., USA), and all tests were two-sided. ANOVA was used to calculate continuous variables and Chi-square was used to calculate categorical variables for baseline characteristics among participants. In the primary analysis, we compared the rate of longitudinal change in baPWV across egg consumption groups. Change in baPWV was calculated as the difference of baPWV measurements (cm/s) between two visits divided by the time interval (years). General linear model regression was used to calculate the adjusted means of baPWV change rate in each group. Model 1 adjusted for age, sex, baseline baPWV, total energy intake, and DASH score; model 2 further adjusted for physical activity, marriage, employment, education level, alcohol use, smoking status, and heart rate; and model 3 further adjusted for systolic blood pressure, FBG, LDL-c, HDL-c. The same model was used to calculate adjusted means of baPWV change rate in each group after further adjusted for other blood pressure indices, DBP, MAP, and PP.

We further tested interactions between egg consumption and covariates in the fully adjusted model.

In the secondary analyses, we examined egg consumption and the risk of developing arterial stiffness during the follow-up among participants who were free of arterial stiffness at baseline. Cox proportional hazard models were used to calculate hazard ratios (HRs) and 95% confidence intervals (CIs), after adjusting for the aforementioned covariates.

## Results

Compared to participants with low egg intake (< 2 eggs/ wk), participants with higher egg intake were more likely to be current smokers, have higher DASH scores and higher degree of education, but lower physical activity (Table 1). There were significant differences in age, total energy intake, and biomarkers crossing every group.

**Table 1:** Baseline characteristics based on egg consumption among 7315 Chinese adults

	Egg consumption					
	0–1.9/wk	2–2.9/wk	3–3.9/wk	4–4.9/wk	≥5/wk	*p* value
	*n* = 325	*n* = 266	*n* = 316	*n* = 5611	*n* = 797	
Age, y	46.5 ± 10.0	48.2 ± 10.5	49.8 ± 10.9	49.0 ± 10.9	46.0 ± 9.6	<.001
Men, %	80.6	71.1	69.0	64.1	81.1	<.001
DASH score	27.3 ± 5.56	28.3 ± 4.61	28.5 ± 4.19	26.1 ± 3.31	28.7 ± 4.73	<.001
Total energy intake, kcal	1590 ± 556	1753 ± 588	1901 ± 467	1638 ± 433	1942 ± 641	<.001
Body Mass Index, kg/m^2^	25.3 ± 3.3	25.4 ± 3.0	24.8 ± 3.1	25.0 ± 3.4	25.0 ± 3.0	0.17
Physical activity						
Low, %	51.9	56.1	50.9	87.9	61.8	<.001
Moderate, %	34.9	40.6	40.6	10.8	32.9	<.001
High, %	13.3	3.4	8.6	1.3	5.3	<.001
Systolic blood pressure, mmHg	134 ± 15.9	136 ± 18.2	136 ± 17.3	130 ± 18.5	135 ± 15.9	<.001
Fasting blood glucose, mmol/L	5.85 ± 1.43	5.94 ± 1.54	6.00 ± 1.72	5.66 ± 1.70	5.95 ± 1.64	<.001
Low-density lipoprotein cholesterol, mmol/L	2.97 ± 0.80	2.99 ± 0.78	3.05 ± 0.86	2.75 ± 0.81	2.96 ± 0.75	<.001
High-density lipoprotein cholesterol, mmol/L	1.37 ± 0.99	1.40 ± 0.47	1.40 ± 0.36	1.35 ± 0.36	1.35 ± 0.60	0.10
Heart rate	74.1 ± 10.7	78.7 ± 11.7	75.7 ± 11.6	74.8 ± 11.6	75.9 ± 10.6	0.08
Married, %	95	100	98.1	95.4	95.7	0.23
Work type, manual, %	84.7	80.3	88.2	79.9	81.9	0.12
Alcohol consumption, %	40.1	36.2	30.7	55.3	30.4	<.001
College education or above, %	8.97	7.94	9.62	18.41	9.57	<.001
Current smoker, %	46.9	39.0	39.7	36.5	47.6	<.001
Baseline baPWV, cm/s	1434 ± 252	1441 ± 288	1438 ± 286	1427 ± 291	1441 ± 265	0.66
Follow-up baPWV, cm/s	1491 ± 284	1503 ± 324	1489 ± 286	1489 ± 334	1503 ± 287	0.82

Abbreviation: *baPWV* brachial-ankle pulse wave velocity

Compared to the participants consuming 0–1.9 eggs/wk., participants consuming 3–3.9 eggs/wk. had the lowest increment in baPWV during follow-up (Table 2). In the final model, the difference in baPWV change rate between this group (adjusted mean: 0.2 ± 11.4 cm/s per year) and the group with consumption of 0–1.9 eggs/wk. (35.9 ± 11.2 cm/s per year) was significant (P-difference = 0.002). Similar results were observed when restricting to participants without arterial stiffness at baseline (Table 2). Further adjustment for other blood pressure indices, such as diastolic blood pressure, mean arterial pressure, and pulse pressure, did not materially change observed results (supplementary Table 1). There was no significant association between egg consumption and the risk of developing arterial stiffness (Table 3). None of the interactions between egg consumption and age, sex, BMI, total energy intake, DASH score, smoking status, alcohol consumption, physical activity level, SBP, LDL, FBG, and heart rate in relation to baPWV change rate were significant (p for interaction≥0.05 for all).

**Table 2:** Adjusted means (± standard error) of baPWV change rate (cm/s/year) by egg consumption group

	Egg consumption					
	0–1.9/wk	2–2.9/wk	3–3.9/wk	4–4.9/wk	≥5/wk	p for trend
n	325	266	316	5611	797	
Model 1	32.2 ± 8.1	20.7 ± 8.9	1.5 ± 8.2*	14.7 ± 2.0	22.6 ± 5.4	0.40
Model 2	38.3 ± 10.9	28.2 ± 11.7	7.0 ± 11.2*	19.4 ± 7.7*	19.4 ± 7.7	0.41
Model 3	35.5 ± 11.2	21.4 ± 12.0	−0.2 ± 11.4*	19.0 ± 8.1	25.0 ± 9.8	0.58
Excluding participants with arterial stiffness at baseline^a^	42.0 ± 12.4	28.7 ± 13.2	10.8 ± 12.6*	24.1 ± 8.7	32.1 ± 10.6	0.77

Model 1 adjusted for age, sex, baseline baPWV, total energy intake, and DASH score

Model 2 adjusted for age, sex, baseline baPWV, total energy intake, DASH score, physical activity, marriage, employment, education level, alcohol consumption, and smoking status, and heart rate

Model 3 adjusted for age, sex, baseline baPWV, total energy intake, DASH score, physical activity, marriage, employment, education level, alcohol consumption, smoking status, heart rate, systolic blood pressure, fasting blood glucose, low-density lipoprotein cholesterol, and high-density lipoprotein cholesterol

Compare to egg consumption 0–1.9/wk., * *p* < 0.05

^a^ Based on model 3

**Table 3:** Hazard ratios and 95% confidence intervals for incident arterial stiffness, according to baseline egg consumption

	Egg consumption					
	0–1.9/wk	2–2.9/wk	3–3.9/wk	4–4.9/wk	≥5/wk	p for trend
Case/total	47/161	41/138	43/158	828/3012	116/396	
Model 1	1	1.01 (0.66,1.54)	0.96 (0.63,1.45)	1.03 (0.76,1.39)	0.94 (0.66,1.33)	0.92
Model 2	1	1.03 (0.67,1.57)	0.99 (0.64,1.51)	1.09 (0.81,1.48)	0.97 (0.69,1.38)	0.92
Model 3	1	1.08 (0.70,1.67)	0.88 (0.56,1.37)	1.12 (0.80,1.55)	0.99 (0.69,1.42)	0.76

Model 1 adjusted for age, sex, total energy intake, and DASH score

Model 2 adjusted for age, sex, total energy intake, DASH score, marriage, employment, education level, alcohol consumption, smoking status, and heart rate

Model 3 adjusted for age, sex, total energy intake, DASH score, physical activity, marriage, employment, education level, alcohol consumption, smoking status, heart rate, systolic blood pressure, fasting blood glucose, low-density lipoprotein cholesterol, and high-density lipoprotein cholesterol

## Discussion

In this longitudinal community-based study, individuals with low (< 2 eggs/wk) vs. high (5+ eggs /wk) egg intake showed similar changes in baPWV and risk of developing arterial stiffness over a mean 3.41 y follow-up. Interestingly, those with egg consumption of 3–3.9/wk. experienced the slowest aging-related increment in baPWV during the follow-up, among all participants, independent of age, sex, overall diet quality, and health status. In this study, we showed an average lower increase in baPWV of 35.7 cm/s per year between the moderate and lowest egg consumption groups, which amounted to ~ 122 cm/s over 3.41 years of follow-up. The difference in accumulated change may be associated with ~ 8% higher risk of CVD, based on a recent meta-analysis [27].

To the best of our knowledge, this study is the first to examine the association between egg consumption and arterial stiffness in a community-based population without CVD. The non-significant difference in baPWV status between two extreme egg consumption categories is consistent with previous systematic reviews and meta-analyses regarding egg intake and mortality, CVD risk, and CVD biomarkers. A recent meta-analysis reported a non-significant difference, for high (7+/wk) vs low (< 1/wk) egg consumption, for death due to ischemic heart disease, ischemic stroke, and hemorrhagic stroke [7]. Similar results were reported in another meta-analysis of egg consumption and total and cause-specific mortality [6]. A systematic review concluded that high-quality intervention studies have found nonsignificant effects of increasing the consumption of eggs on risk markers for CVD and type 2 diabetes in healthy subjects and type 2 diabetic subjects [27]. Another two recent meta-analyses of randomized controlled trials also reported non-differential effects of egg consumption (> 4/wk) on inflammation [9], blood pressure [8], and lipid profiles [8] compared to egg consumption ≤4 eggs/week. In contrast, a recent pooled study based on 6 US cohorts reported that higher egg intake was associated with higher CVD incidence and all-cause mortality [10]. However, there was no significant difference when comparing individuals who consumed < 1 egg/d with those with no egg consumption [10].

We found that participants with modest egg consumption (3–3.9/wk) had a significantly lower age-related increment in baPWV across all egg consumption groups. Of note, when the binary variable of arterial stiffness was used as the outcome, a similar trend was observed, but it became non-significant. Several previous studies have reported a low risk of CVD and mortality being associated with modest egg consumption. For example, in a recent prospective study including 146,011 individuals from 21 countries, those who consumed 3–4.9 eggs/wk. had the lowest risk of mortality and CVD across all egg consumption groups [28]. In this study, compared to egg consumption < 1 egg/wk., 3–4.9 eggs/wk. had lower risks of mortality and CVD. In contrast, the difference in risk of mortality and/or CVD was not significant for 5 + eggs /wk. vs < 1 egg/wk. [28]. In a prospective study in Sweden, individuals consuming 3–6 eggs/wk. had the lowest risk for heart failure, myocardial infarction, and hemorrhagic stroke, but not ischemic stroke, across all egg consumption groups, although the difference was not significant [29]. A similar non-significant U-shaped trend was also observed for the association between eggs and total mortality [4, 5, 27, 30].

Arterial stiffness is the stiffening of the blood vessel wall. When the blood vessel loses its elasticity, its ability to control the blood flow is compromised. The blood vessel needs more force to push blood through, which increases the pulse pressure and may introduce damage to the blood vessels [31]. Eggs, as a type of nutrient-dense food, are an accessible source of L-arginine, vitamin D, vitamin K, choline, and xanthophyll carotenoids, specifically lutein and zeaxanthin [3], which have been shown to be protective against arterial stiffness. The amino acid, L-arginine, is a precursor of nitric oxide. Nitric oxide inhibits the expression of adhesion and further improves endothelial function [32, 33]. Endothelial dysfunction is a risk factor for arterial stiffness [34, 35]. Therefore, modest egg consumption might be potentially protective from endothelial dysfunction and arterial stiffness. Xanthophyll carotenoids, lutein and zeaxanthin, and choline have been shown to decrease inflammation [36–38]. However, choline is also a source for trimethylamine-N-oxide, which has been shown to be associated with a high risk of atherosclerosis and other cardiovascular events [39, 40]. On the other hand, eggs are a major source of dietary cholesterol and saturated fat [3]. A previous meta-analysis showed that high egg consumption was associated with elevated serum cholesterol, [4] a potential risk factor for arterial stiffness [41, 42]. High saturated fat intake has also been associated with high aortic pulse wave velocity [43]. Given both positive and negative nutritional components in eggs, it is not surprising that our result, and others, appears to be non-linear, with modest, but not low, egg consumption associated with the desired health outcome. However, further studies are needed to confirm this association.

However, given the lack of consistency in findings, more research is needed. As a majority of existing studies were conducted in western countries, more investigations at various geographic areas are necessary. This will be beneficial for future meta-analyses.

This study has some limitations. Egg consumption was assessed with a single self-reported question, which may introduce measurement error (misclassification) or recall bias. Undifferentiated misclassification could bias toward the null. In this study, total energy intake was likely to be underestimated due to the small number of food items in the food frequency questionnaire, which might miss some important foods. Thus, total energy intake was adjusted as a covariate in all multiple regression models in order to reduce the influence of this systematic error. Because this study is a preliminary study, the follow-up (mean 3.41 years) is relatively short, which is another limitation. Because the majority of participants were in the 4–4.9 eggs/wk. group, the sample size in the extreme categories was small, which may limit the statistical power to detect small-to-moderate effects. The study population is localized, and the observed results may not generalize to other ethnic groups with different eating habits and social economic backgrounds. Only 2406 women were included in the analysis and the menopausal status was not assessed in our questionnaire, which is also one of the major limitations.

## Conclusions

In conclusion, in this large-scale longitudinal analysis, we did not find a significant difference in arterial stiffness, as assessed by baPWV level, between low and high egg consumption groups (0–1.9/wk. vs. ≥ 5/wk). However, moderate egg consumption (3–3.9 eggs/wk) appeared to have beneficial effects on arterial stiffness. Further studies, investigating the association between egg consumption and arterial stiffness with longer follow-up years and more women participants, are warranted to replicate our findings.

## Supplementary Information

**Additional file 1: Supplementary Table 1.** Adjusted means (± standard error) of baPWV change rate (cm/s/year) by egg consumption group in Chinese adults, after further adjusted for DBP, MAP and PP.

## Data Availability

The datasets used and analyzed during the current study are available from the corresponding author on reasonable request.

## Abbreviations

CVD: Cardiovascular diseases
PWV: Pulse wave velocity
baPWV: Brachial-ankle pulse wave velocity
mHEI-2015: Modified healthy eating index 2015
IPAQ: International physical activity questionnaire
FBG: Fasting blood glucose
HDL-c: High-density lipoprotein cholesterol
LDL-c: Low-density lipoprotein cholesterol
HRs: Hazard ratios
CIs: Confidence intervals
T2D: Type 2 diabetes
DBP: Diastolic blood pressure
MAP: Mean arterial pressure
PP: Pulse pressure
