# Supplementary material for: Association between egg consumption and arterial stiffness: a longitudinal study
Source: Nutr J. 2021 Jul 13;20:67. doi: 10.1186/s12937-021-00720-6 (PMC8278728; doi:10.1186/s12937-021-00720-6)
Supplement: Supplementary file 1 — Additional file 1: Supplementary Table 1. Adjusted means (± standard error) of baPWV change rate (cm/s/year) by egg consumption group in Chinese adults, after further adjusted for DBP, MAP and PP. [file 12937_2021_720_MOESM1_ESM.docx]

**Supplementary table**

**Supplementary table 1. Adjusted means (± standard error) of baPWV change rate (cm/s/year) by egg consumption group in Chinese adults, after further adjusted for DBP, MAP and PP**

|  | Egg consumption | | | | |  |
| --- | --- | --- | --- | --- | --- | --- |
|  | 0-1.9/wk | 2-2.9/wk | 3-3.9/wk | 4-4.9/wk | ≥5/wk | p for trend |
| n | 325 | 266 | 316 | 5611 | 797 |  |
| Multivariate model* + DBP | 36.7±11.2 | 22.8±12.0 | 1.9±11.5* | 19.0±8.1* | 26.5±9.8 | 0.53 |
| Multivariate model* + MAP | 36.7±11.2 | 22.8±12.0 | 1.9±11.5* | 19.0±8.1* | 26.5±9.8 | 0.53 |
| Multivariate model* + PP | 36.7±11.2 | 22.8±12.0 | 1.9±11.5* | 19.0±8.1* | 26.5±9.8 | 0.53 |

* Adjusted for age, sex, baseline baPWV, total energy intake, DASH score, physical activity, marriage, employment, education level, alcohol consumption, smoking status, heart rate, systolic blood pressure, fasting blood glucose, low-density lipoprotein cholesterol, and high-density lipoprotein cholesterol.

Abbreviation: DBP, diastolic blood pressure; MAP, mean arterial pressure; and PP, pulse pressure.
